# Nurture, Pleasure and *Read and Resist!*: Abolition Feminist Methodology for a Collective Recovery?

**DOI:** 10.1007/s10691-021-09463-5

**Published:** 2021-05-31

**Authors:** Felicity Adams, Fabienne Emmerich

**Affiliations:** School of Law, Keele University, Newcastle, UK

**Keywords:** Abolition feminism, Black lives matter, Care, COVID-19, Erotic, Resistance

## Abstract

COVID-19 has magnified intersecting inequalities that are central to the functioning of capitalism. At the height of the crisis, the value of an economy based on the exchange of goods and services faded away to expose the importance of care across the public and private spheres. Undervalued and underpaid labour suddenly became critical to the survival of many. Drawing on Abolition Feminism, we argue for the need to seize this revaluation of labour to centre nurture and pleasure within our post-pandemic recovery. We apply an Abolition Feminist framework that conceptualises the prison as part of a network of violence that deflects attention from the root causes of harm. We reflect on the development of our Abolition Feminist web platform, *Read and Resist!*, a space where theory meets reflection on praxis. We consider how activist strategies within Abolition Feminism may support us in reimagining our relationships with law and justice post-COVID-19.

## COVID-19, Black Lives Matter, and Care

Like everyone else we were hit by this pandemic

first not wanting to believe,

being stuck at home with our ‘loved ones’ in a situation that we had never contemplated—with

parents, with a small child, with a visitor who was otherwise homeless

In the immediacy we had to adjust to the lack of space,

the new confined environment and

at the same time trying to make sense of COVID-19 and all the uncertainty around it.

Strong emotions and numbness were part of our everyday experiences; feeling a sense of guilt and helplessness towards beautiful people who were falling ill, who were trapped by this illness and those who lost their lives.

The images of those who were dying were multiplying and

we began to notice that the vast majority of care and key workers impacted by this illness were Black or from ethnic minority backgrounds

We were separated and felt isolated, detached from each other

and we felt alone

stuck in cramped spaces trying to keep afloat

We were lucky, we had work and a home; meanwhile the government announced the expansion of police and immigration officer powers effectively to “manage” the vulnerable

What brought us together was a return to basics

reading as a collective, as part of our reading group *Read and Resist!*

Together along with Forough, Jane, Sophia, Debbie, Alex, and Sorcha.

we covered a variety of areas from the Feminist Judgments Project, law and emotion, carceral feminism, Abolition Feminism, and survival

We felt the need to dismantle the frame of reference set by mainstream narratives of the pandemic that deflect attention from structural violence

clapping, drawing pictures, and appropriating rainbows

Public appreciation to care workers, key workers, retail workers, delivery drivers and warehouse workers—putting their lives on the line…

Is not matched by a political and economic revaluing of care …

Instead the government announced a prison expansion programme

to build ourselves out of this pandemic

with inherently violent sites in which racism, homophobia, transphobia, and ableism thrive

A smokescreen…

Meanwhile the number of deaths keep rising -

the anonymity of the dead increasing; especially those from Black or ethnic minority backgrounds.

All until the murders of so many including

Breonna Taylor, Tony McDade, Ahmaud Arbery, and George Floyd

When the leaders of the Black Lives Matter movement and fellow protestors uttered “No justice, no peace!”

And united against systemic racism, anti-blackness, homophobia, transphobia and coercive institutions

And pledged to build more liveable societies for all people.

Fuelled by the courage and creativity of the BLM protestors in the face of multilayered crises and a desire to take action and find a collective pathway to recovery

We felt compelled to break out

and transcend the crisis—drawing inspiration from the Black Lives Matter movement and our reading group’s discussion of Abolition Feminism to create *Read and Resist!*

## Rejecting the ‘New Normal’: Decentring the Coercive Force of Law

We wrote our reflective impressions in July 2020 to capture our emotions at the time.^1^ In the public revaluation of labour during the pandemic, we recognised an opportunity to reposition nurture at the core of our collective recovery strategies.^2^ As prison abolitionists, we understand that central to this vision must be a vision of society that is free of coercive institutions such as the prison. Yet, the government’s reflex response is to fund violent institutions such as prisons and the police that perpetuate a myriad of systemic inequalities and structural violence (Adams and Emmerich 2021). In doing so, the government prioritises the expansion of existing individualised carceral responses that fail to address the root causes of harm, rather than seizing this moment to invest in vital community-led initiatives that centre nurture, healing and pleasure (Olufemi 2020; Adams and Emmerich 2021). This is something that can and must be challenged.

We wanted to move beyond critiquing neoliberalism to focus on seeking and building communities centred on nurture and pleasure. We draw on Abolition Feminism as a reflective and conceptual framework to anchor both our academic and activist work. For this, we reconnect with Black feminist roots that point us to “the development of integrated analysis and practice based upon the fact that the major systems of oppression are interlocking” (Combahee River Collective [1977], 2017, 15).^3^ Abolition Feminism shows us that we can and must centre nurture and pleasure in our post-pandemic recovery. Abolition Feminism teaches us that to foreground pleasure and nurture, we must simultaneously decentre prisons and the police and re-centre people’s material needs including mental health, housing and community support. In the current socio-political context, we recognise that this is a challenging and complex process. However, as Ruth Wilson Gilmore asserts, “crisis means instability that can be fixed only through radical measures, which include developing new relationships and new or renovated institutions out of what already exists” (Gilmore 2007, 26). The current conjuncture of crises demands a radical reimagining of carceral measures to enable us to deeply engage with the multifaceted nature of our social worlds and to build new nurturing ways of being that bypass the “carceral creep” in our post-pandemic lived realities (Kim 2020, 253–254).^4^

The process of reflection led us to create a multifunctional web platform, *Read and Resist!* (www.read-and-resist.org) to bring together and amplify grassroots voices marginalised in the mainstream academic discourse. We seized on the rupture created by the current moment to craft an online space where theory meets reflection on praxis.^5^ We set out to build a collective and collaborative Abolition Feminist forum bringing together a host of community, activist and academic voices on all things Transformative Justice.^6^ Before we move onto our reflections on *Read & Resist!*, we briefly situate this piece conceptually in Abolition Feminism. This is followed by an overview of *Read and Resist!* as both a space for reflecting on nurture, healing and pleasure as strategies to move forward and a space for amplifying marginalised knowledges.

## Transcending the Prison: The Transformative Potential of Abolition Feminism

Abolition Feminism can be understood as both a framework for imagining alternative futures and a methodology and praxis. It is a “complex, anti-colonial, anti-racist, anti-capitalist revolutionary approach to change” (Davis in Incite! 2020) and an interrogative methodology and praxis centred on responding to the root causes of violence through revaluing care (Olufemi 2020).

Abolition Feminism is about envisioning alternative transformative strategies to the carceral status quo that challenge the conditions under which violence in all forms is permitted to exist and thrive. It means grasping this present chapter in the intersecting crises to actively engage in complex conversations about race, gender, class, sexuality and ability in order to revolutionise our conditioned responses to interpersonal violence and resist the trap of the carceral web. As part of this, we must all consistently ask ourselves, as Angela Davis suggests:

“How easy it is to reach for existing strategies and tools assuming that they alone can bring about change… racism and repression are not discreet problems that can be removed by dissection but rather are integrally woven into the very fabric of carcerality.” (Incite! 2020).

In her pioneering book *Are Prisons Obsolete?*, Angela Davis suggests that we understand the prison as integrated into a “set of relationships that comprise the prison industrial complex” (Davis 2003, 106). Conceptualising the prison as part of a network of interconnected coercive systems can help us think about alternatives to prison as a process of decarceration across a spectrum of different spaces (e.g., schools, health, physical and mental care). In effect, new institutions could “crowd out” the “prison industrial complex” (Davis 2003, 106). In the work to “crowd out” the prison and re-centre nurture and pleasure, Abolition Feminists have paired anti-violence and prison abolition agendas both to engage with the systemic harm of interpersonal violence and to transcend carceral responses to violence.

Ruth Wilson Gilmore (2007), in her ground-breaking book *Golden Gulag: Prisons, Surplus, Crisis, and Opposition in Globalizing California*, sets the foundations for Abolition Feminist activism. She writes about the California prison expansion and its effects on the spaces that are linked to incarceration either through labour or the absence of sons, daughters, brothers, sisters, fathers and mothers. She highlights the power of community activism, through the work of the Mothers Reclaiming Our Children, that rejects incarceration and reclaims their loved ones and their community spaces centred on care and nurture. She highlights the importance of supporting agency at the community level, which can create the foundation for transformative justice activism. We have seen with Black Lives Matter how meaningful actions in multiple places that are amplified via social media can, as Gilmore (2007) puts it, “shake the ground. In other words, movement happens” (248).

In similar and divergent ways, these contributions show us that Abolition Feminism stages a crucial intervention in the existing terrain in a way that engages deeply with trauma and violence and foregrounds justice. Levine and Meiners (2020) develop the nexus established between trauma, violence and justice under Abolition Feminism. They emphasise our collective freedom from violence as contingent on our freedom to experience healing, pleasure and nurture (Adams 2020).

### Abolition Feminism: Pleasure Activism and Nurturance Culture

We understand interconnection between justice, liberation, nurture and pleasure is central to Abolition Feminism and is reflected in activist scholarship on ‘nurturance culture’ and ‘pleasure activism’. Nora Samaran (2019), in her book *Turn This World Inside Out: The Emergence of Nurturance Culture*, sets out in conversation with others to explore ways for us to strive as a collective, to grow our skills for care, empathy, and in turn justice (Samaran 2019, 21). Through the inversion of violence as nurturance, Samaran opens up ways to think differently about our responsibilities, particularly as white people with privilege, to take responsibility to both challenge oppressive structures, and to heal ourselves and others without burdening people from marginalised communities. Samaran shows us that justice can be reimagined as community care where we are accepted as our whole selves, where those harmed are kept safe, and where the person who has harmed is challenged to empathise and repair. To cultivate a “nurturance culture”, we need to transcend shaming responses and replace them with a “healthy remorse” (Samaran 2019, 128–129). This will open up our propensity for empathy so that we can “Own. Apologize. Repair” (Samaran 2019, 166). Tapping into the public revaluation of labour with a focus on nurture, we understand that we can create a model of justice that embraces our interconnectedness and that holds us to account free from shame where our whole selves are recognised. This creates an environment where the harmed person feels safe to call out the harm and to find empathy.

Similarly, in her book, *Pleasure Activism: The Politics of Feeling Good*, Adrienne Maree Brown (2019a) illustrates how pleasure is central to realising our true power, liberating ourselves from the systemic harms produced under these structures and feeling justice as a transformative experience (16). Brown roots her conception of pleasure in Audre Lorde’s *Uses of the Erotic: The Erotic as Power*, which urges us to yield the full depth of our desires as a “replenishing and provocative force” for transformation (Lorde [1978], 2019a, 40). Brown encourages us to regain our “whole… selves” flattened under neoliberal capitalist structures in order to reclaim freedom as a deeply satisfying experience (Brown 2019a, 19). This extends to harm and trauma. She conceives the prison as a cog in the “vicious systems that have been normalized” (Brown 2019a, 16). Brown encourages us to transcend “mediocrity” (Lorde [1978] 2019a) offered by surface-level criminal justice responses to harm and to engage with the true depth of our experiences. This is pleasure activism in action. Ultimately, we will “begin to understand the liberation possible when we collectively orient around pleasure and longing” (Brown 2019a, 4). Understanding this within the context of nurturance culture, we can take risks, be vulnerable in the knowledge that we are accepted as whole and thereby work towards social and political change. We see tapping into pleasure and nurturance culture in all its forms as particularly important now, because under the conditions of the pandemic our lives have been upended. From our readings of* Pleasure Activism* and *Nurturance Culture*, we sought to resist the toxicity of neoliberal, capitalist structures and tapped into our sensuality to imagine an alternative way of living and working together, one that centres “learning, playing, practicing, doing things anew” (Brown 2019a, note 13).

## “We felt compelled to break out—to transcend the crisis …”: *Read and Resist!*

The idea for *Read and Resist!* as an Abolitionist Feminist collective and collaborative forum came to us in June 2020, as a culmination of our experiences of the pandemic and the Black Lives Matter movement. It followed an initial feminist reading group that Flick (Felicity) had started in March 2020 and coincided with the first lockdown. The reading group created space and time for us to read and to meet with each other. After a while, we realised that we were looking to move beyond the immediate concerns and fears brought about by the pandemic and what we recognised as its neoliberal iterations centred on the normalisation of lockdown and strategies of individualisation and responsibilisation. We wanted to move towards a more creative and critical space beyond academia for both reflection and building community in response to the trauma of the pandemic and the systemic inequalities exposed by it.

We were inspired by the revaluation of care and nurture in public discourse to craft *Read and Resist!* as an online space where theory meets reflection on praxis. Building on Abolition Feminist and Black Feminist calls for collective and community-centred activist strategies that transcend neoliberal and coercive ways of being and living, we created a multifunctional web platform to bring together and amplify knowledges marginalised in mainstream academic discourse and to create a space for reflecting nurture and pleasure in response to harm and trauma. The aim is to build a collective and collaborative forum that connects a host of community, activist and academic voices on all things Transformative Justice. As part of the web platform, we have developed a monthly open digital reading group (*Read and Resist!*), a blog (*Write and Resist!*), a podcast *(Listen and Resist!*) and a YouTube Channel (*Watch and Resist!*). These spaces come together under the overarching title of the web platform: *Read and Resist!*.^7^ Together these activities offer two interlocking spaces which we will outline in the following subsections: a space for reflecting on nurture, healing and pleasure, and a space for amplifying marginalised grassroots knowledges.

### A Space for Reflecting on Nurture, Healing and Pleasure

In our experience, the COVID-19 pandemic and the resulting measures have magnified stress and anxiety. They have further entrenched systemic inequalities, because the burdens of care labour and precarity are disproportionately carried by marginalised groups. We felt that managerialist strategies that centre on surveillance and the rationing of information have created a work environment, located in the home during the first lockdown, in which we were made to feel guilty, or shame for experiencing pleasure. For those of us who are privileged to be in employment, we have found that we were not working from home, but ‘sleeping at work’.^8^ Following the end of the first lockdown, during the summer of 2020, we found that the expansion of neoliberal, patriarchal and heteronormative governance strategies were part of our lived experience, forming the ‘new normal’. We felt subsumed into new digital spaces that quickly became all-encompassing experiences, filling our days with a myriad of notifications of mundane activities. At the same time, our impression was that the sick and the dead were increasingly dehumanised and became statistics in the public discourse. We felt a desperate need to be part of and to co-create a nurturing, healing, pleasurable space to ‘crowd out’ these normalising processes. This was reflected in the contributions to the reading group discussions and has become a continuing thread across all of the reading group sessions. The reading group has become a space where theory meets reflection on praxis.

The reconfigured *Read and Resist!* monthly reading group went digital in October 2020 and brought together participants (students, activists and academics) from Keele, the US and Europe to discuss pleasure activism, nurturance culture and joy. The reading group provided an opportunity through collective reading and discussion to re-centre a reflection on nurture and pleasure in our daily lives and to disrupt the normalisation of an extreme and painful situation. Members of the group came with different levels of experience in transformative justice. For us and our co-participants, the reading group provided a designated time and space to learn from each other outside of mainstream work and to think about how we embed nurturing and pleasurable strategies as part of our post-COVID 19 recovery strategies. During our discussions, we were reminded by one of our contributors of the need to focus on “dismantling carceral logic in interpersonal relationships and communities”.^9^ This means engaging with our collective fallibility and resisting moral judgments. This requires us to actively transcend shame and guilt and replace it with a “healthy remorse” (Samaran 2019, 128–129). This is not a simple process, but it is essential for creating a nurturance culture that centres pleasure. Drawing on Mariame Kaba’s activism, another of our contributors highlighted the centrality of failure in discovering new and nurturing avenues for liberation.^10^ We recognise that learning and making mistakes can render us vulnerable to value judgments on all sides. However, in reality, making mistakes, as another contributor highlighted, empowers us to think about “how we create space or situations where we feel more alive”.^11^ We felt a need to create a space where we were able to engage with the multidimensional nature of our feelings and ourselves, including our incompleteness, where people can be themselves among others and trust in the knowledge that others will not judge them. From comments in the space, messages and tweets, we get a sense that there is a degree of mutuality of experiences among co-participants. One of our postgraduate students reflected on her feelings of anger and helplessness towards global injustice in a blog piece in which she highlights the need to be a part of collective action towards change (Martin 2020).

### A Space for Amplifying Marginalised, Grassroots Knowledges

In addition to the reading group, as part of the web platform, the blog (*Write and Resist*!), podcast (*Listen and Resist*!) and YouTube Channel *(Watch and* *Resist*!) provide a space for students, people with lived experiences, activists and academics to express themselves true to their personal positionality and to be heard free from encumbrances and academic writing conventions.^12^ We understand this framework for amplifying knowledges outside of the mainstream academic discourse as central to allyship against racism, transmisogyny, homophobia, misogyny, ableism, classism and xenophobia.^13^ We follow the call by both Samaran and Brown, who unsettle how the burden of care and the labour of harm reduction are appropriated and remind us to challenge practices and discourses that place the burden of change onto marginalised communities (Samaran 2019; Brown 2019b). It is for us, as white people with privilege, to relieve the burden from those who are marginalised to expose harms, violence and trauma produced under “the imperialist, white supremacist capitalist patriarchy” and to deal with the aftermath (hooks 2013, 36). Through a focus on nurture and pleasure, we can work towards the reduction of personal and systemic harm collectively without resorting to shame, guilt and punishment central to the functioning of the prison (Samaran 2019).

Here we share some initial insights with reference to some of the blogs we have published as part of* Write and Resist!* since September 2020. Contributors have shared experiences positioned across multiple intersections and covering a diverse range of topics, such as racism, policing and education; power and decolonising the curriculum; and transphobia, racism and policing.^14^ The blog aims to open up a space for vital interventions in our everyday lives.

Olanrewaju Paul Olubayo (2020) challenges us to keep the momentum going following the unprecedented summer of resistance. He says it is up to us as white allies to step up and support the Black Lives Matter movement. He encourages us to reflect on our personal behaviours and actions in reinforcing harmful and toxic structures: “Above all, are you ensuring that every day you exist in a manner which actively opposes the white supremacist superstructure and fights for Black Lives?” He provokes us to take stock and at the same time encourages us to be personally accountable. This aligns with Abolition Feminism that teaches us that these struggles demand that people with privilege relieve the burden placed on marginalised communities (Samaran 2019).

Abolition Feminism’s “anti-colonial, anti-racist, anti-capitalist revolutionary approach to change” (Davis in Incite! 2020) has prompted us to promote an editorial process that facilitates and promotes decolonised writing. This is to challenge the paradigms that govern the way we write in academia. We find academic writing conventions are largely underpinned by systemic exclusionary practices and standards that perpetuate westernised, heteronormative and ableist systems of power. Forough Ramezankah’s blog breaks out of traditional academic writing convention to explore the power of autoethnography (2020). She highlights how in education, which should be a nurturing space, asymmetric power relations are produced through privileging of the dominant language. For her, autoethnography “can be a tool to decolonise writing, it can be a transformative tool to collectivise individuals, it can encourage collaboration between people who are marginalised also between those who are marginalised and those who are privileged” (Ramezankah, 2020). She encourages us to embrace this method and share it with our students to open up possibilities for encountering and being open to the Other. For her, the writing process in this space was a form of refuge, a healing experience.^15^

Similarly, Mike Duffy resists the ableist confines of academic writing conventions to explore his lived experiences of the enforced isolation under COVID-19 for a person living with a severe degenerative disease. He writes a stream of consciousness, including emojis and colloquialisms to reflect on how he is both produced as potentially undeserving under the DWP and inherently vulnerable by the Ministry of Health during the pandemic (Duffy 2021). We see retaining idiolect as a vital part of amplifying the depth of Duffy’s experiences, as a marginalised person during the current moment:

“Well, that was just funny as fuck…for 15 years I have to prove I’m ill… describe my most personal information and in great detail, to get my benefits, yet suddenly the government knows I’m extremely vulnerable wtf... it really was one of those moments” (Duffy 2021).

In our opinion, mainstream academia often seeks to contour writers’ voices. We feel Abolition Feminism provides a disruption to neoliberal culture which suppresses our collective freedom to be heard as our whole selves.^16^ We understand writing as a fluid process entwined with our lived experiences. Such writing has the potential to disrupt hegemonic discourses and to open a space for reimaginings to be possible.

## Seeking and Building Communities as a Way Forward

We have experienced neoliberal pressures to maintain an undisturbed level of productivity in our daily working lives which has forced us to eschew and flatten the material realities presented by the pandemic. They have prevented us from engaging fully with our feelings and experiences in the current moment. Simultaneously, this has limited the radical potential of what can be constructed outside of these dominant structures during this period of instability (Gilmore 2007, 26). While many conceive of abolition as the tearing down of institutional spaces, Ruth Wilson-Gilmore reminds us that “abolition is not absence, it is presence. It’s about building life-affirming institutions” (Gilmore cited in Nicholls 2017). Guided by the vision of Abolition Feminism, *Read and Resist!* aims to create nurturing, pleasurable spaces that centre marginalised knowledges to help us collectively reimagine toxic structures and build transformative and just futures. *Read and Resist!* recognises that:

“Abolition is a hopeful vision that means each moment where harm happens is an opportunity to transform relationships and communities, build trust and safety, and grow slowly toward the beautiful people we are meant to be, in the world that we deserve” (Shank 2020, 33)

Through *Read and Resist!*, we are decentring the law, as well as building a space for amplifying counter-conducts and community-centred activist strategies that transcend neoliberal and coercive ways of being and living (Smart 1989, Foucault cited in Dean 2010, 21). In developing *Read and Resist!* during the current conjuncture of crises, we aim to seize the transformative potential offered by Abolition Feminism, nurture and pleasure in the hope that we too will craft the societies that we desire and deserve.

Abolition Feminists reinforce nurture, healing and pleasure as foundational to imagining an alternative future. Reflecting on our experiences of the pandemic and the lockdown, we have learnt to begin with ourselves and how we engage with one another in our daily lives and work. From our understandings of Abolition Feminism, we recognise nurturing of the self as vital in the process of exploring and building sustainable futures. A focus on nurturance and pleasure urges us to create spaces in which we are safe to feel all our emotions—joy, anger, anxiety—but also where we can be our whole selves, take risks without fear of vulnerability, and reposition pleasure and the erotic to the fore of our collective recovery and liberation strategies (Lorde 1978).

## Footnotes

1 We adopt the first-person pronoun “we” throughout this reflection to refer to our personal experiences as co-authors unless otherwise specified.

2 We are not the only ones who have thought of this, see The Care Collective (2020).

3 The statement is reproduced in chapter 2 of Taylor (2017).

4 As survivors of sexual assault we understand that we cannot address complex harm and trauma with coercive and oppressive structures that play into our isolation and reproduce systemic harms.

5 We see this as rooted in feminist and anti-capitalist, radical publishing practice.

6 To clarify, Abolition Feminism takes an intersectional approach and is inclusive of all identities.

7 We decided to retain the title of the original reading group, *Read and Resist!*, as the title for our developed web platform and reading group to enable us to open up the space through a variety of interconnected mediums including “writing”, “listening” and “watching”.

8 Sleeping at work is a comment we heard on BBC Radio 1.

9 Anonymised comment, 14.10.2020.

10 Kaba, Mariame Twitter post, June 2020, 4.20 pm, https://twitter.com/prisonculture/status/1272548582139330566.

11 Anonymised comment, 14.10.2020.

12 We appreciate that people occupy multiple social locations and standpoints.

13 See the In Conversations with Maya Schenwar and Victoria Law and Abolitionist Lawyer, Kushal Sood Write and Resist. *Write and Resist!*. Accessed March 26th 2021, https://read-and-resist.org/write-and-resist-2/.

14 See https://read-and-resist.org/write-and-resist-2/.

15 Personal comment to the authors.

16 This is reminiscent of Hélène Cixous écriture féminine.
